# Intramuscular lipoma of the subscapularis muscle: Lipoma intramuscular no músculo subescapular

**DOI:** 10.1590/1516-3180.2014.1321537

**Published:** 2014-02-01

**Authors:** Débora Balabram, Carla Cristina de Sousa Resende Cabral, Omar de Paula Ricardo, Cristóvão Pinheiro de Barros

**Affiliations:** I MD. Doctoral Student, Department of Anatomical Pathology and Legal Medicine, School of Medicine, Universidade Federal de Minas Gerais (UFMG), Belo Horizonte, Minas Gerais, Brazil; II MD. Radiologist, Serviço de Radiologia e Ultrassonografia de Minas Gerais (Sermig), Belo Horizonte, Minas Gerais, Brazil; III MD. Pathologist, Laboratory of Anatomical and Diagnostic Pathology, Belo Horizonte, Minas Gerais, Brazil; IV MD. Breast Surgeon, Instituto da Previdência dos Servidores do Estado de Minas Gerais (IPSEMG), Belo Horizonte, Minas Gerais, Brazil

**Keywords:** Lipoma, Rotator cuff, Axilla, Diagnosis, differential, Magnetic resonance imaging, Lipoma, Bainha rotadora, Axila, Diagnóstico diferencial, Imagem por ressonância magnética

## Abstract

**CONTEXT:**

Intramuscular lipomas are benign tumors that infiltrate the muscles.

**CASE REPORT:**

We describe the case of a 58-year-old female patient with an axillary lump. The lump was a lipoma inside the subscapularis muscle. It is important to differentiate these lesions from liposarcomas and from other diseases that may present as axillary lumps. The most accurate imaging method for differentiating benign lipomatous tumors from liposarcomas is magnetic resonance imaging, but surgical removal of these intramuscular lesions to confirm the diagnosis is recommended.

**CONCLUSION:**

Intramuscular lipomas are a rare cause of benign axillary lumps and should be considered in making differential diagnoses on axillary masses.

## Abstract (pt)

**CONTEXTO:**

Lipomas intramusculares são tumores benignos que infiltram os músculos.

**RELATO DE CASO:**

Descrevemos o caso de uma paciente de 58 anos com nódulo axilar. O nódulo era um lipoma na intimidade do músculo subescapular. É importante diferenciar essas lesões de lipossarcomas e outras doenças que podem acometer a axila. O método de imagem mais eficaz para diferenciar lesão lipomatosa benigna do lipossarcoma é a ressonância magnética, mas é recomendada a remoção cirúrgica dessas lesões intramusculares para confirmar o diagnóstico.

**CONCLUSÃO:**

Lipomas intramusculares são causas raras de nódulos axilares benignos e devem ser considerados no diagnóstico diferencial dessas lesões.

## INTRODUCTION

Intramuscular lipomas are benign tumors that infiltrate the muscles.^1^ They are larger than superficial lipomas and are most common in the lower extremities and trunk.^1^ ^,^ ^2^

We report the case of a 58-year-old patient with a painless axillary lump and discuss possible diagnoses. 

## CASE REPORT

**Figure 1:**
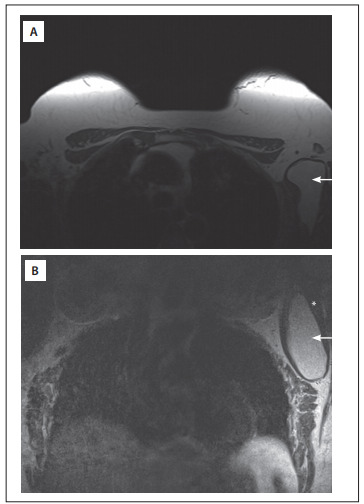
Magnetic resonance imaging. A: T1-weighted axial image; B: T2-weighted coronal image. Arrow, intramuscular lipoma. Asterisk, subscapularis muscle.

A 58-year-old woman visited the breast disease clinic of the Public Servants' Social Security Institute of the State of Minas Gerais (Instituto da Previdкncia dos Servidores do Estado de Minas Gerais, IPSEMG) in November 2010 and reported a lump. On clinical examination, she was found to have a left axillary lump with hard consistency, close to the border of the *latissimus dorsi* muscle. The cytological analysis (using material obtained through an ultrasound-guided procedure) suggested that this was a lipoma. Magnetic resonance imaging (MRI) showed a lesion in the left axilla suggestive of a lipoma inside the subscapularis muscle (Figure 1). 

**Figure 2:**
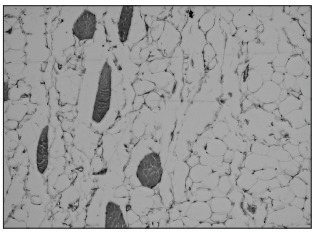
Mature adipocytes with no nuclear abnormalities; muscle fibers within the lipoma.

In March 2011, the patient underwent surgery to remove the lesion, and the pathological examination confirmed the hypothesis of an intramuscular lipoma (Figure 2), measuring nine centimeters.

## DISCUSSION

Intramuscular lipomas are an entity comprising slowly growing benign tumors that infiltrate the muscles.^1^ They have been called infiltrating lipomas. It is important to differentiate them from liposarcomas and, in the axillae, from other axillary diseases (such as lymph node infiltration due to malignant, infectious and immunological diseases).^3^ ^-^ ^6^ Specifically, in this case, a thorough investigation of the breast was carried out to rule out carcinoma. Nowadays, the most accurate imaging examination for differentiating a benign from a malignant lipomatous tumor is magnetic resonance imaging. Infiltration of the muscle bundles, homogenous appearance, lack of peripheral capsule and presence of few fine, regular septa distinguish benign lipomas from liposarcomas.^1^ ^,^ ^2^ ^,^ ^7^ ^,^ ^8^ Surgical removal and histological examination should be performed after imaging of the lesion, since neither method is infallible.^1^ ^,^ ^2^ ^,^ ^9^ ^,^ ^10^

We found some case reports in PubMed, Lilacs and Embase, reporting lipomas located in the rotator cuff (Table 1), but none of them was located in the subscapularis muscle.^11^ ^-^ ^14^

**Table 1:**
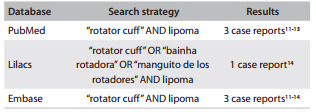
Case reports retrieved from the review of the medical databases. Search date: February 28, 2013

## CONCLUSION

Intramuscular lipomas are a rare cause of benign axillary lumps and should be considered in making differential diagnoses on axillary masses.
